# A Proposed Decision-Making Framework for the Translation of In-Person Clinical Care to Digital Care: Tutorial

**DOI:** 10.2196/52993

**Published:** 2024-06-26

**Authors:** Anna DeLaRosby, Julie Mulcahy, Todd Norwood

**Affiliations:** 1 Physera Physical Therapy Group, San Francisco, CA, United States; 2 Omada Health, San Francisco, CA, United States

**Keywords:** clinical decision-making, digital health, telehealth, telerehab, framework, digital medicine, cognitive process, telemedicine, clinical training

## Abstract

The continued demand for digital health requires that providers adapt thought processes to enable sound clinical decision-making in digital settings. Providers report that lack of training is a barrier to providing digital health care. Physical examination techniques and hands-on interventions must be adjusted in safe, reliable, and feasible ways to provide digital care, and decision-making may be impacted by modifications made to these techniques. We have proposed a framework to determine whether a procedure can be modified to obtain a comparable result in a digital environment or whether a referral to in-person care is required. The decision-making framework was developed using program outcomes of a digital physical therapy platform and aims to alleviate barriers to delivering digital care that providers may experience. This paper describes the unique considerations a provider must make when collecting background information, selecting and executing procedures, assessing results, and determining whether they can proceed with clinical care in digital settings.

## Introduction

### Background

Digital health is revolutionizing health care, and the COVID-19 pandemic has led to rapid acceleration of the use of digital health technologies, particularly the adoption of telehealth. Digital health, including the use of telehealth or telemedicine, allows health care practitioners to provide services without being in the same physical location as the patient. Telehealth can include synchronous or asynchronous messaging with providers, video calls, audio-only calls, and the secure transmission of information over the internet between patients and their providers [1]. Digital health can also include information gathered by medical devices, wearable sensors, apps, or other software [2]. The application of technology in health care has a vast potential to increase access to care and improve quality.

Research indicates that telehealth outcomes are equivalent to in-person care in rehabilitation [3-5] and can be an effective intervention for addressing pain and function limitations in a variety of musculoskeletal conditions [6]. Clinical outcomes from telehealth episodes of care are comparable with in-person rehabilitation for conditions such as osteoarthritis, low-back pain, hip and knee replacement, multiple sclerosis, and cardiac and pulmonary rehabilitation [3]. Increasing evidence supports that telehealth physical therapy delivered by a mobile app provides clinical outcomes comparable with those of in-person care [3,4,7]. Research also reveals that telehealth decreases travel time and costs [8]. It is well documented that patients recognize the benefits of telehealth as well, demonstrating high engagement [9-11] and high levels of satisfaction across multiple metrics, including quality of care, convenient access to multiple specialists, improved care and coordination with digital care, and outcomes similar to in-person care [12-17].

Despite evidence of the benefits of telehealth, there are barriers to the integration of telehealth into traditional health care models. For example, physical therapists (PTs) report apprehension toward utilizing telehealth in their practice, reporting insufficient preparation and inadequate knowledge about how to implement telehealth visits, influencing providers’ acceptance, preferences, and outcomes [12,18,19]. Further, less than half (42%) of health care providers surveyed believed telehealth was as effective as face-to-face care, and 21% reported insufficient training [18,19]. Another significant barrier to digital health adoption is the belief that lack of physical contact hampers accurate diagnosis and management [12,18,19]. Successful integration of telehealth into traditional health care models will only be achieved through addressing provider beliefs about the efficacy of telehealth and instruction in providing equivalent care through a new model.

Telehealth requires the translation of traditional clinical skills to a new medium [20,21]. Remote patient care is characterized by dynamic patient environments, unique safety concerns, and a lack of traditional patient care tools, forcing the provider to act in new and dynamic ways to provide effective care. When encountering new clinical scenarios, many providers look for guidance through decision-making frameworks. Frameworks outline a structured and systematic approach to problem-solving that incorporates evidence and specific context, and promotes informed decisions [22]. When used in health care, decision-making frameworks can ensure consistency, reduce bias, and enhance the quality of decisions and quality of care [22-24]. A standardized process assists health care professionals in assessing risks and benefits, improves outcomes, and provides patient-centered evidence-based care [24].

Delivering effective care in a digital health setting requires that health care providers adapt their thought processes to account for the nuance of the interactions between technology and the patient to enable sound clinical decision-making in the digital health setting. This paper introduces a decision-making framework to determine whether a clinical procedure is feasible in a telehealth setting with similar quality, accuracy, and reliability as in-person encounters, or when the use of an equivalent but alternative procedure is most appropriate. We propose that utilizing a clinical decision-making framework can alleviate clinicians’ concerns about the efficacy of digital health and assist the implementation of clinical best practices in a digital setting. The purpose of this paper is threefold: (1) to propose a decision-making framework to train and inform health care providers that increases provider efficacy with the translation of skills to this new medium; (2) to propose a thought model that allows quantitative testing through implementation research; and (3) to realize the potential for telehealth for patients and providers to improve access to care independent of geography.

### Development of the Framework

This framework was the result of a review of the current literature and the authors’ combined expertise in providing telehealth physical therapy. The authors have a combined 18 years of experience in telehealth, including providing patient care, designing and implementing training for providers, as well as managing a nationwide network of telehealth PTs. This framework has been applied to clinical practice and refined based on the outcomes of over 10,000 patient cases.

Analysis of program outcomes and the identification of PT behaviors that lead to positive clinical outcomes influenced the development of this framework. Program data confirmed that provider behavior during telehealth episodes directly impacts clinical outcomes in an app-based telehealth physical therapy program [4] and that when interventions provide high value, patients will be highly engaged [11] resulting in cost savings [25]. Prior literature describes how to translate specific evidence-based evaluation techniques for the application of telehealth and how to utilize established clinical practice guidelines for telehealth episodes [26-32]. However, procedure-specific training cannot prepare providers for the dynamic nature of telehealth encounters that include variations in the patient’s environment, health status, caregiver support, digital literacy, equipment availability, and other factors. In response to the ever-changing context of telehealth visits and to fully equip health care providers working in a digital environment, a decision-making framework was developed. This framework was designed to help providers identify the relevant factors in the clinical picture, assess possible actions, and make decisions that lead to positive clinical outcomes. The process of defining this framework was iterative, data-driven, and emphasized patient-centered design. We incorporated an understanding of the users on our platform, the tasks they completed, and the digital environment; development was driven and refined by patient surveys, feedback, and outcomes. We believe this framework will assist clinicians in translating their clinical skills to digital practice to enable optimal clinical outcomes, convenience, and satisfaction. Initially, learning to leverage the steps of the framework may increase time in decision-making but as the clinician becomes experienced the process will become efficient and give more options for the telehealth environment.

### Utilizing the Framework

Appropriate application of a decision-making framework in a clinical setting requires that certain conditions are met. First, the clinical problem must be within the scope of the clinician’s practice. This ensures the clinician is appropriately trained and licensed to provide care and make clinical decisions. In the case of digital health, appropriate training includes proficiency with digital tools, technology, and website manner in addition to medical or clinical training [33,34]. Second, the patient must be appropriate for digital care. Appropriateness for care requires that the patient’s cognition level, medical status, digital literacy, communication abilities, technology access, physical environment, and preference all support safe digital care interactions. Finally, providers must consider the security and regulatory implications of digital care, including ensuring compliance with HIPAA (Health Insurance Portability and Accountability Act), local and federal privacy regulations, and data security requirements. If the provider, patient, and technology all meet these conditions, the application of this framework is appropriate.

**Figure 1.**
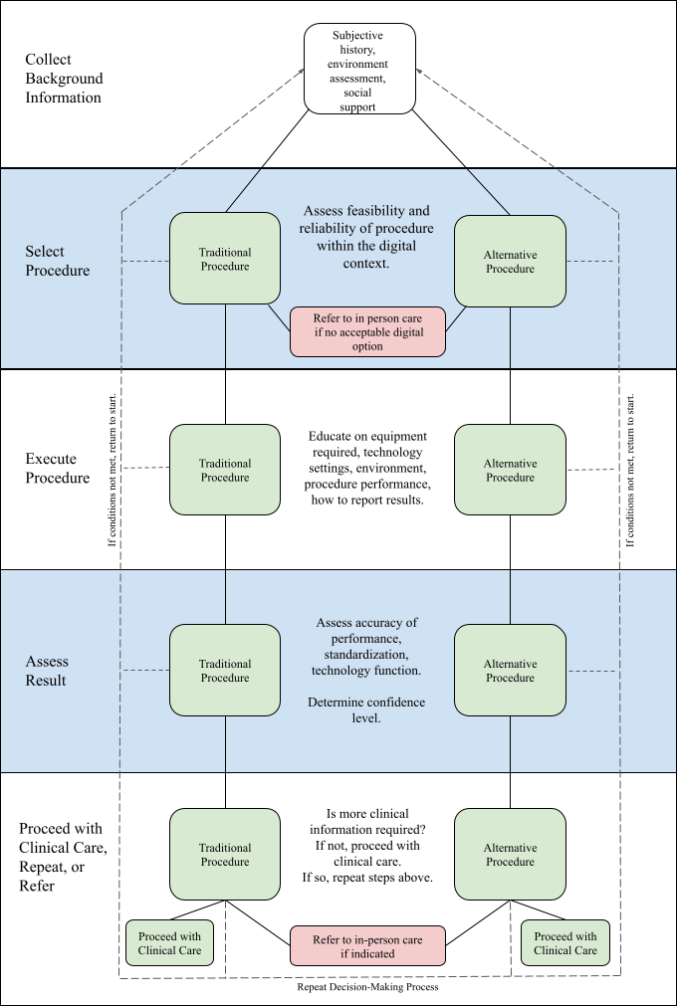
The decision-making path. At every step of the patient encounter, providers must determine whether telehealth is the best option for the clinical scenario. The determination process should be the same whether the provider is using a traditional procedure or a procedure that has been modified for the patient’s environment. At each step, the provider must determine whether they can continue down the decision-making path, or if they need to return to the start of the decision-making process using an alternative procedure. If no acceptable digital option exists at any step, they must refer to in-person care.

At each step of the process, the provider must determine whether telehealth is the most appropriate method of providing care. When a provider determines that telehealth is not appropriate for the patient, they should inform the patient of the next steps, which may include activation of emergency services, coordination of care to facilitate referral to a specialist, in-person visit, or obtaining labs or imaging. Figure 1 provides a visual representation of the steps included in this decision making framework.

## Description of the Decision-Making Framework

### Step 1: Collect Background Information

Clinicians may collect relevant clinical information using data from chart review and review of a digital intake form. The subjective interview of a telehealth visit should proceed as it does in an in-person visit, with emphasis on the chief complaint, relevant health history, current and past medical conditions, and social history. The subjective portion of the examination may also include a visual assessment of the patient’s environment, inquiry about equipment availability, and availability of caregiver support, which are factors unique to telehealth but enhance the clinical picture. If at the conclusion of the subjective interview, the provider has identified an urgent medical need, or that telehealth is not appropriate then the patient may be referred to in-person care at this time. If the provider is confident that they have collected the information needed to inform the objective examination and that it is safe and appropriate to continue with a telehealth objective examination, they will move to the next step.

### Step 2: Select an Examination Procedure

Providers will select the examination procedures based on the information gathered in the subjective examination. Procedures should be evidence-based and relevant to the differential diagnosis process. Once a procedure has been selected, the provider must consider the feasibility, reliability, and validity of the procedure when performed in a digital setting.

To evaluate feasibility, we consider whether the patient has the resources, space, ability, and knowledge necessary to complete the procedure safely. The provider will consider information gathered in the subjective portion regarding the patient’s cognitive status, physical ability, social support, environment and technological resources, and time available to determine if the procedure can be accurately performed. If the setup for a test is complicated or the instructions are lengthy, the time constraints of a patient visit may make a test not feasible.

Reliability is the quality of a measure that produces reproducible scores on repeat administrations of a test. Reliability is thus a prerequisite for test validity [35]. Validity is the measure of how accurately a test measures the underlying trait of interest [35,36]. When assessing patients in-person, reliability is supported by a clinical environment standardized for all sessions. In digital health settings, tests are performed in the patient’s environment and providers must look for alternative ways to ensure results are reliable and valid. If a traditional procedure cannot be performed with acceptable feasibility and reliability, then providers should consider if an alternative procedure can provide the same clinical information. Alternative methods will be unique to the patient’s resources, abilities, and environment, but alternatives should be assessed for feasibility and reliability. Functional testing is often an acceptable alternative for traditional tests when the equipment or environment is standardized.

The reliability of functional tests can be increased if the same equipment in the home is used for subsequent testing. For example, a 30-second sit-to-stand test [37] using the same chair in the patient’s home will give a clinician reliable data for each assessment. Further, measurements such as joint range of motion, can be tracked by having the patient reach to low, medium, or high shelves in their home and reassessed using the same shelves. This technique allows the provider to monitor and document progress in an easily accessible, functional and standardized way within the patient’s environment.

Selecting a procedure means that the provider will make dynamic decisions unique to the patient they are seeing. For example, during an in-person visit, manual muscle testing of internal rotation of the shoulder is often used to indicate subscapularis muscle rupture or dysfunction. In digital settings, the provider cannot provide manual resistance, but the same information can be obtained using the Gerber test [38]. If the patient is unable to achieve the testing position for a Gerber test, a provider could consider functional strength testing such as lifting canned goods. In this scenario, the provider will ensure reliability by using the same number of cans at each assessment. To ensure validity, the provider must ensure that the patient is performing the test correctly; in this example, a patient lifting the canned goods with a straight arm would provide an invalid result but lifting with a bent elbow would appropriately stress the biceps and give a valid result.

If there is no procedure that can be performed that is feasible and reliable in the digital setting, and this information is required for clinical decision-making, then a referral to in-person care would be indicated. For example, if a clinician suspects rupture of the anterior cruciate ligament and determines that a Lachman test is necessary, but is not feasible via telehealth, then a referral for in-person assessment is required.

### Step 3: Execute the Clincial Procedure

Performing the clinical procedures in digital settings requires different skills than in in-person settings. Digital settings require the provider to assist the patient in managing their environment and any relevant equipment needed during the visit. Therefore, it is incumbent on the provider to communicate with the patient explicitly about the procedure prior to execution and ensure they have the relevant equipment and can use it appropriately.

The provider should communicate what equipment is needed (eg, a sturdy chair with arms). Providers should give clear directions to the patient on how to set up any equipment and where the patient should be positioned. Additionally, the provider must describe how to utilize technology during the procedure. Appropriate audio, video, and lighting setup ensures the provider can see and hear the patient adequately while they perform the tasks. The provider should review each step of the procedure with the patient prior to performing it and allow the patient to ask questions or clarify instructions. The patient should have a good understanding of what information the procedure is gathering so that the patient can monitor and report the appropriate variable during the procedure. For example, during a balance assessment, the patient should understand if they are balancing for as long as they can without toe touches, or if they should count the number of toe touches within the given time frame. The provider should document the method used for the procedure, equipment, setup, and outcome to ensure subsequent tests can be performed in a standard way. If the patient is unable to perform the procedure as directed by the provider, then the provider should consider alternative procedures or referral to in-person care.

### Step 4: Assess Results

Once the procedure has been performed, the provider determines whether the result answers the original clinical question and their confidence level in the result. Confidence will be affected by how accurately the patient was able to follow the provider’s instructions, and if technology worked as expected. If the patient performed the test incorrectly or if there was video or audio lag or poor clarity available, the provider may have low confidence in the result. A procedure that was performed as instructed in an environment that was reliably standardized using the same equipment and set up with technology that worked without disruption will provide high confidence.

### Step 5: Proceed With Clinical Care, Repeat, or Refer

High confidence in the outcome allows the provider to continue care in the digital setting. If the provider has low confidence in the result, they can repeat steps 1 through 4 again using an alternative procedure to achieve a result that provides high confidence. If the provider is seeking information that is essential to the care of the patient and no procedure can be performed in a manner that provides a result that is reliable, reproducible, and yields high confidence, then a referral to in-person care is needed. Table 1 provides a list of the factors that should be considered when making clinical decisions in digital settings.

**Table 1.** The relevant factors the provider should consider as they progress through the decision-making process. At each stage, the provider must determine whether telehealth is appropriate for this clinical scenario.

Factors	Key points
Collect background information	Subjective history may include chief complaint and health history as well as: Cognition level Digital literacy Communication abilities Technology access Features of physical environment Patient preference for digital health tools If each criterion is not met, then the patient must be referred to in-person care
Select procedures	Traditional procedures, digital alternatives, or functional tests may be used if they are: Necessary for clinical reasoning Evidence-based Feasible Reliable If no procedure meets these criteria, then the patient must be referred to in-person care
Execute procedures	Instruct the patient about: Equipment required Technology settings Environment set up Performance of the procedure Outcome reporting If execution of the procedure is impeded by any of these factors, the provider will consider alternative procedures or refer to in-person care
Assess results	Determine if the reliability of the result was affected by: Procedure performance Technology Reporting accuracy Does the provider have confidence in the result of the procedure?
Proceed with clinical care, repeat, or refer	Do you need more clinical information? If no: Proceed with clinical care If yes: Repeat decision-making steps Refer if no alternative exists

### Clinical Application

#### Overview

**Figure 2.**
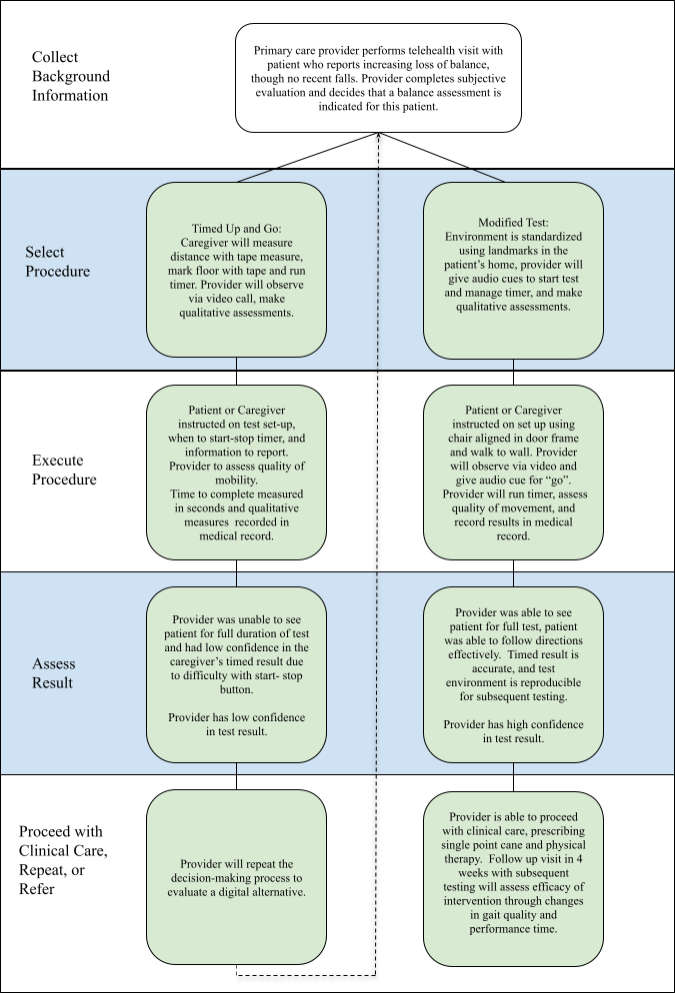
Description of clinical application of proposed decision-making framework using the timed up and go test and modified test. The provider proceeded through the first process but had low confidence in performance. They then repeated the decision-making process with modifications made to the test environment and procedure. The modified test produced a high-confidence result and allowed clinical care to proceed.

The application of this decision-making framework can be illustrated through clinical examples. This example provides descriptions of how procedures can be modified but provides high-value clinical information when feasibility, reliability, and reproducibility are considered. Assessment of confidence allows providers to determine the value of the result prior to proceeding with clinical care or referring to in-person care. Figure 2 provides a visual representation of the decision-making process used in the patient scenario.

#### Patient Scenario

Consider a hypothetical case of a 79-year-old woman living in a rural community who scheduled a telehealth visit with her primary care provider (PCP) to discuss concerns regarding mobility. Mobility assessment is within the scope of the provider in this example, who has the appropriate training and expertise to perform telehealth visits. The visit will take place on the platform provided by the health system and meet applicable HIPAA and data security requirements. The provider has access to the patient’s medical history as a part of the software platform.

#### Step 1: Collect Background Information

The patient’s chief complaint is frequent stumbling, often the result of catching her toe while walking, which has resulted in loss of balance, frequently holding onto furniture or walls while walking, and avoiding walking in the community due to fear of falling. She reports no falls to the ground and no other health status changes but is concerned that her balance will continue to decline. The provider assesses the patient’s cognitive status, communication ability, and preference for digital health during the subjective assessment. As part of the telehealth visit, the provider completes red flag screening and review of systems and finds no neurological deficits, no indication of cardiac impairment, and no history to suggest that the mobility concerns are the result of sinister pathology. Her PCP would like to quantify the mobility impairments in a standardized way during the telehealth visit and the patient agrees to this. The patient reports that her husband is available during the telehealth visit to assist if needed. As the patient has no current history of falls, health history is clear, and the patient has a caregiver present, the PCP feels confident that they can complete a mobility assessment safely via telehealth.

#### Step 2: Select Procedure

The PCP chooses the Timed Up and Go (TUG) test [39] as it is evidenced-based and recommended by the Center for Disease Control STEADI protocol [40]. TUG is a timed mobility test in which patients rise from a standard chair, walk to a line on the floor 10 feet away, turn, return to the chair, and sit. Patients are instructed to wear their regular footwear and can use a walking aid during the test if needed.

The PCP assesses feasibility by asking if the patient has access to the equipment needed: a sturdy chair such as a dining chair, stopwatch, tape measure, and tape. The PCP describes the test to the patient and husband and asks if they feel able to achieve the setup and execute the test. The PCP will be able to gather qualitative information about gait during the test by having the patient face their device’s camera toward the test area. The outcome of the TUG is time-based, which the PCP determines to be reliable through digital means. The PCP decides that the caregiver will manage the stopwatch to mitigate any lag in the internet connection during the test. Using a tape measure to define distance and using the same chair in the same location will ensure that the test setup is reproducible for subsequent testing. The provider educates the patient’s caregiver on the start or stop timing procedure of the TUG, further ensuring reliability. The PCP will assess qualitative mobility by visually assessing movement during the test using the camera of the mobile device. The PCP determines that the TUG is feasible and reliable in a digital setting and provides the clinical information required to make clinical decisions about treatments for this patient, so no alternative is necessary.

#### Step 3: Execute Procedure

The PCP instructs the patient’s husband to gather a sturdy chair and stopwatch, measure 10 feet on the floor, and mark it with a line of tape. The provider instructs the patient and caregiver to arrange the camera of their mobile device in a manner that allows the PCP to observe the test. The caregiver is instructed on starting or stopping the stopwatch. The patient is instructed on the test procedure according to the standard TUG instructions. The caregiver is instructed to report the time to completion of the procedure to the PCP. The provider answers clarifying questions for the patient and caregiver, and they perform the test. During the test the provider can hear that the caregiver fumbles with the stopwatch, and the patient leaves the video frame.

#### Step 4: Assess Results

While the environment setup was standardized supporting reliability, the caregiver reported difficulty with starting or stopping the timer, impacting the accuracy of the timed result. The patient left the visual frame during the test, impacting the ability to assess qualitative aspects of gait such as stopping and changing directions. The provider determines they have low confidence in the result and is unable to determine if the patient exceeded the recommended time of <12 seconds for test completion, or if there are mobility deficits that prompt recommendations for assistive device use.

#### Step 5: Proceed With Clinical Care, Repeat or Refer

The provider has low confidence in the result of the test and does not feel they can proceed with clinical care based on the results. The need for mobility assessment remains, and the provider feels that modifications of the testing scenario may allow them to gain the clinical information they need, so a referral to in-person care is not necessary. The home environment had only one area where a 10-foot space was available to complete the TUG, however, the family was unable to position the camera in a manner that allowed the whole area to be seen by the provider. Additionally, the caregiver had difficulty starting and stopping the timer, decreasing the accuracy of the result. The provider determines that the variables measured by the TUG test appropriately provide the clinical information they need, but he will need to utilize an alternative testing method to enable him to address the limitations. He will repeat decision-making steps using a digital alternative to gain the information he needs from the mobility assessment.

Background information remains the same, so the provider can proceed to select an alternative procedure. They decide to address the limitations of the first attempt by choosing a new testing location where they can standardize the test using landmarks in the patient’s home. The caregiver is instructed to position the front legs of the chair even with a door frame and will have the patient walk to the end of the hallway, touch the wall, and return to the chair. The distance walked is less than the 10 feet required of the TUG, but the patient is visible to the provider the whole distance. Additionally, the provider will give audio cues to start and stop the test while he manages the timer remotely. The provider and patient determine together that this setup is feasible and easily reproducible for subsequent testing. The modifications will allow the provider to assess movement quality as well as ensure timed results are accurate, which addresses the limitations of the prior test.

Execution of the modified procedure requires instruction regarding chair location and placement of the mobile device so the camera captures the whole testing area. The patient is instructed on how to perform the modified test procedure. The performance of the modified test proceeds without audio or video lag or distortion. After the second test provider feels confident that the timed result was successful. The provider was able to assess the quality of mobility throughout the whole test. Because the provider has high confidence in the clinical information they obtained through the alternative test, they can proceed with clinical care. The provider determines that the patient would benefit from using a single-point cane to improve balance with changing directions when walking. The PCP also prescribes physical therapy to address balance, gait, and lower extremity strength. The patient will schedule a follow-up telehealth visit with the PCP in 4 weeks and they will repeat the modified mobility test at that time using the same setup to assess the effect of these interventions.

## Discussion

### Principal Findings

Providing a decision-making framework for clinicians to utilize in digital care can alleviate clinicians’ concerns about implementing digital care in their practice. To our knowledge, a framework that assists providers in translating in-person clinical skills to digital care does not exist. This framework enables clinicians to practice effectively in the most accessible environment for the patient while prioritizing evidence-based practice, assessing risks, and providing patient-centered care. As digital care is increasingly desired by patients [19,23,41,42], it is imperative that providers are confident in decision-making in digital settings so telehealth remains safe, efficient, and equivalent to in-person care.

The value of a clinical procedure is reliant upon the feasibility, reliability, and clinician confidence, as well as the interaction of those variables with digital technology. A procedure that is feasible, reliable, and reproducible, but is performed poorly and provides low confidence has less value in clinical decision-making than an alternative digital procedure that deviates from standard performance but instills high confidence in clinical decision-making. This improves patient safety by determining whether a patient can remain in a digital care environment or should be referred to in-person care. Additionally, the framework encourages clinicians to use evidence-based practice guidelines as the basis for care, modifying procedures in a feasible and reliable manner to improve outcomes. This will ensure consistency, reduce bias, and enhance the quality of decisions in digital care [22-24]. The application example demonstrated that modifications made based on the patient’s environment and technology limitations enabled the provider to proceed with digital care in a manner consistent with clinical best practices and supported the provision of safe, effective, and quality care.

Time is a valuable resource in medical care, and providers must be confident in decisions made during clinical encounters. In situations where decisions must be made quickly, utilizing a framework can assist with decision-making efficiency [22-24]. Novice clinicians or providers who are transitioning to digital care may benefit from a framework to help them determine the best course of action in a timely manner. With increased provider experience and repetition, the decision-making process will be more efficient and timelier. For example, experienced telehealth clinicians become proficient in scanning the patient environment, determining feasibility based on available resources, as well as becoming efficient at modifying traditional procedures based on the patient’s environment, and instructing patients on camera setup and how to utilize technology efficiently. In scenarios like the clinical application described above, an experienced provider may identify potential barriers prior to execution and decide to utilize a modified procedure from the start to save time.

This framework builds on the existing literature that shows similar diagnostic accuracy between in-person and digital examination techniques [26,29-31]. Lack of physical contact when working through telehealth was perceived to hamper accurate and effective diagnosis and management [18]. However, many commonly performed physical examination techniques have poor sensitivity and interrater reliability. This is evident in the poor interrater reliability scores of techniques such as palpation of lumbar structures [43] and assessment of breath sounds [44]. Decision-making tools that enable providers to evaluate alternative methods for gathering clinical information help to overcome these barriers and increase confidence that practitioners are providing effective, safe care. Additionally, adapting procedures allows patients the full benefit of telehealth, including convenience, cost-savings, better adherence, higher engagement, and improved access to care in rural or underserved areas [20].

### Future Research

Avenues for further research should include randomized control trials comparing trained versus untrained providers to determine whether the utilization of this framework leads to improved clinical outcomes, provider self-efficacy, and patient satisfaction scores, and would provide insight to overcoming the barriers to digital health that providers may experience. Research is needed in implementation science to determine if training clinicians in using a framework will increase treatment fidelity. Similarly, this framework can be considered in future studies to provide further evidence of the efficacy of digital care and enable the full potential of telehealth for all stakeholders.

Further understanding of how providers make decisions to include digital tools in patient care is needed. Understanding provider confidence in modifying in-person techniques and clinical problem-solving in digital settings may improve providers’ willingness to utilize digital care with their patients. Provider training about how to modify traditional procedures, evaluating the efficacy of modified procedures, and assessing confidence in results may increase provider self-efficacy in digital settings. Best practices and standardized education for health care providers on how to effectively use digital tools should be established.

### Limitations

There are limitations of this framework as it is broad in scope and cannot address every situation. Independent tests should be performed to evaluate the usability of the framework and its effectiveness in improving guideline implementation. We recognize that no single framework can be used for all guidelines or contexts. Provider behavior will be influenced by environment, resources, technology, and other factors despite training in using a decision-making framework.

## Conclusion

We created a framework for clinicians to determine whether a particular procedure can be performed feasibly in a digital health setting with the same quality, accuracy, and reliability as in a traditional setting. Utilizing a framework to assist in clinical decision-making is important to alleviate clinicians’ concerns about using digital tools and help guide the translation of the best available evidence from traditional care to digital care. The increased demand by patients for digital care requires a new set of clinical skills, and this framework enables providers to comply with clinical best practices and offer high-quality care for patients who want to receive their care via telehealth.

## Abbreviations

HIPAA: Health Insurance Portability and Accountability Act
PCP: primary care provider
PT: physical therapist
TUG: Timed Up and Go
